# New *Topoisomerase I *mutations are associated with resistance to camptothecin

**DOI:** 10.1186/1476-4598-10-64

**Published:** 2011-05-27

**Authors:** Céline Gongora, Nadia Vezzio-Vie, Sandie Tuduri, Vincent Denis, Annick Causse, Céline Auzanneau, Gwenaëlle Collod-Beroud, Arnaud Coquelle, Philippe Pasero, Philippe Pourquier, Pierre Martineau, Maguy Del Rio

**Affiliations:** 1 IRCM, Institut de Recherche en Cancérologie de Montpellier; INSERM, U896; Université Montpellier1; CRLC Val d'Aurelle Paul Lamarque, Montpellier, F-34298, France; 2 INSERM U916, Institut Bergonié & Université de Bordeaux, Bordeaux, France; 3 INSERM, U827, Montpellier, F-34000 France, Université Montpellier1, UFR Médecine, Montpellier, F-34000 France; 4 Institute of Human Genetics CNRS UPR1142, F-34396 Montpellier, France

## Abstract

**Background:**

Topoisomerase I (TOP1) is a nuclear enzyme that catalyzes the relaxation of supercoiled DNA during DNA replication and transcription. TOP1 is the molecular target of camptothecin and related drugs such as irinotecan and SN38 (irinotecan's active metabolite). Irinotecan is widely used as an anti-cancer agent in the treatment of metastatic colon cancer. However, its efficacy is often limited by the development of resistance.

**Methods:**

We previously established several SN38 resistant HCT116-derived clones to study the mechanisms underlying resistance to SN38. Here, we investigated whether resistance to SN38 in these cell lines could be linked to the presence of *TOP1 *mutations and changes in its expression and activity. Functional analyses were performed on these cell lines challenged with SN38 and we specifically monitored the double strands breaks with γH2AX staining and replication activity with molecular combing.

**Results:**

In SN38 resistant HCT116 clones we identified three new *TOP1 *mutations, which are located in the core subdomain III (p.R621H and p.L617I) and in the linker domain (p.E710G) and are packed together at the interface between these two domains. The presence of these *TOP1 *mutations in SN38 resistant HCT116 cells did not modify TOP1 expression or intrinsic activity. Conversely, following challenge with SN38, we observed a decrease of TOP1-DNA cleavage complexes and a reduction in double-stranded break formation). In addition, we showed that SN38 resistant HCT116 cells present a strong decrease in the SN38-dependent asymmetry of replication forks that is characteristic of SN38 sensitive HCT116 cells.

**Conclusions:**

These results indicate that the *TOP1 *mutations are involved in the development of SN38 resistance. We hypothesize that p.L617, p.R621 and p.E710 TOP1 residues are important for the functionality of the linker and that mutation of one of these residues is sufficient to alter or modulate its flexibility. Consequently, linker fluctuations could have an impact on SN38 binding by reducing the enzyme affinity for the drug.

## Background

Irinotecan (CPT-11), a semi-synthetic water-soluble derivative of camptothecin, is widely used for the treatment of metastatic colon cancer in first- and second-line therapies [1]. CPT-11 is a pro-drug which is converted by carboxylesterases into the active form SN38. Like other camptothecin derivatives, SN38 exerts its cytotoxic activity through inhibition of Topoisomerase 1 (TOP1). Human TOP1 is a nuclear enzyme responsible for the relaxation of supercoiled DNA, which is needed for DNA replication, transcription and chromatin condensation [2,3]. TOP1 first introduces a nick in one strand of duplex DNA and then religates the TOP1-linked DNA break. SN38 interferes with TOP1 activity by inhibiting the religation step and induces the formation of stable covalent ternary complexes at DNA breakage points [4]. As a consequence, collision with the replication machinery produces double strand breaks at the replication fork [5].

The most frequently reported cellular mechanisms of resistance to CPT-11 include reduced intracellular drug accumulation (mediated by ABC transporters) [6,7], alterations in CPT-11 and SN38 metabolism [8], quantitative and qualitative alterations of the TOP1 protein [9-11] and alterations in the cellular response to ternary complex formation that ultimately lead to repair of DNA damage or cell death [3,12].

*TOP1 *mutations that confer resistance to camptothecin derivatives have been identified in mammalian cells and yeast [13-16]. Most of them are located close to the active site of the enzyme or clustered in two regions of the core domain [17]. Recently, Benedetti and colleagues showed that the p.A653P mutation limits the flexibility of the linker domain [18]. Studies of clinical specimens are needed to determine whether such mutations can be found also in patients and are involved in chemotherapy resistance. Among the few studies that have investigated the presence of *TOP1 *mutations in clinical samples [19-21], only one reported two point mutations (p.W736X and p.G737S on the same allele) in tumor tissues from a patient treated with CPT-11 [21]. However, this result has never been confirmed.

We have previously established SN38 resistant clones from the human colon carcinoma cell line HCT116 to investigate the mechanisms that lead to resistance to SN38 [7,22]. In this study, we identified three new *TOP1 *mutations in these clones. Moreover, we show that, following treatment with SN38, DNA cleavage complexes and DNA double strand break formation are reduced in SN38 resistant cells as well as the SN38-induced asymmetry of the replication fork that is typical of SN38 sensitive cells. Finally, the localization of these new *TOP1 *mutations suggests that they could influence the linker flexibility and possibly alter TOP1/SN38 interaction.

## Methods

### Cell lines

**Figure 1:**
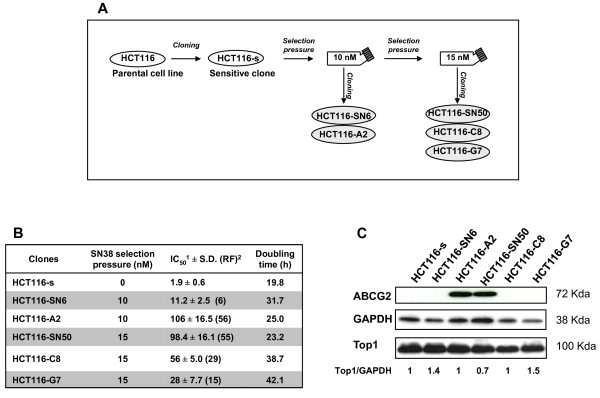
**Generation and characterization of SN38-resistant HCT116 cells**: A) Schematic representation of the production of SN38-resistant HCT116 cell clones. B) Drug sensitivity and growth rate of the HCT116 clones: IC_50 _values were determined using the Sulforhodamine B assay; the resistance factor (RF; in between brackets) was determined by dividing the IC_50 _value of each resistant clone by that of the sensitive clone HCT116-s. Data represent the mean ± SD of at least 3 independent experiments. Doubling time was calculated during the exponential growing phase as following: doubling time (in hours) = h*ln(2)/ln(c2/c1); c1 and c2 are the cell concentration at the beginning and the end of the chosen period of time C) TOP1 and ABCG2 protein expression by western blotting. Equal loading is shown by GAPDH. Values below the blot represent relative quantization of TOP1.

The HCT116 colon adenocarcinoma cell line was purchased from ATCC (Manassas, VA, USA). Cells were grown in RPMI 1640 supplemented with 10% fetal calf serum (FCS) and 2 mM L-glutamine at 37°C under a humidified atmosphere with 5% CO_2_, and passaged by trypsinization. The HCT116-s and HCT116-SN6, HCT116-A2, HCT116-SN50, HCT116-C8 and HCT116-G7 clones were obtained as previously described [7]. Briefly, parental HCT116 cells were first cloned to obtain a reference SN38 sensitive clone, referred to as HCT116-s. This sensitive clone was then continuously exposed to SN38 with stepwise increased concentrations ranging from 1 nM to 15 nM over a period of approximately 8 months. The cell population growing in 10 nM SN38 was cloned to obtain the HCT116-SN6 and HCT1116-A2 clones, and the cloning of the population growing in 15 nM SN38 gave us the HCT116-SN50, HCT116-C8 and HCT116-G7 clones (Figure 1A). Drug-selected clones were maintained in the appropriate concentration of SN38. All the cell lines were cultured in drug-free medium at least 5 days prior to any experiment.The HCT116-SN6 and HCT116-SN50 cells have been previously described and partially characterized [7,22].

### Drug sensitivity assay and calculation of the doubling time

Cell growth inhibition and cell viability after SN38 treatment were assessed using the Sulforhodamine B (SRB) and the clonogenic assays as previously described [22]. To calculate the doubling time (DT), cells (15 ×10^4^) were seeded in seven identical 25 cm^2 ^flasks for each counting experiment. Then, every 24 hours the viable cells of one flask were counted with a hemocytometer after Trypan blue staining. DT was calculated during exponential growing phase as following: DT = h*ln(2)/ln(c2/c1); h = time point in hours; c1 and c2 = cell concentration at the beginning and at the end of h.

### Immunoblotting

Whole cell lysates were prepared as previously described [22]. Proteins from the extracts (10^5 ^cells per lane) were electrophoretically separated on 10% SDS-PAGE, then electro-transferred onto polyvynilidene difluoride membranes (Amersham Biosciences, Buckinghamshire, UK). Membranes were incubated with the appropriate primary antibody: polyclonal anti-Top1 [23], anti-ABCG2 [7] anti-GAPDH (clone 6C5) antibody from Millipore (Billerica, MA, USA),. Secondary antibodies were peroxidase-conjugated anti-mouse (Sigma-Aldrich, St. Louis, MO, USA) and anti-rabbit anti-sera (Sigma-Aldrich, Saint-Louis, MO, USA).

### Nucleotide sequencing

Total RNA was isolated from sensitive and resistant HCT116 cells using the RNeasy Mini Kit (Qiagen, CA, USA), followed by synthesis of first-strand cDNA. *TOP1 *cDNA was amplified by PCR in 6 overlapping fragments, using the following primers:

(1) 5'-CTCAGCCGTTTCTGGAGTCT-3' and 5'-TCAGCATCATCCTCATCTCG;

(2) 5'-CGAAAAGAGGAAAAGGTTC-3' and 5'-GGGCTCAGCTTCATGACTTT;

(3) 5'-CCACCATATGAGCCTCTTCC-3' and 5'-CCTTGTTATCATGCCGGACT;

(4) 5'-AGAGCCTCCTGGACTTTTCC-3' and 5'- GACCATCCAACTCTGGGTGT-3';

(5) 5'- TTCGTGTGGAGCACATCAAT-3' and 5'- GACCTTGGCATCAGCCTTAG-3';

(6) 5'-CGAGCTGTTGCAATTCTTTG-3' and 5'- ACCACACTGTTCCTCTTCAC-3'.

Purified PCR products were then sequenced using the same sense primers by the dideoxynucleotide method using the MWG Biotech sequencing service.

### *Top1 *cDNA cloning

The *TOP1 *region that contains the p.R621H and p.E710G mutations was PCR amplified using the 5'-AGAGCCTCCTGGACTTTTCC-3' and 5'- ACCACACTGTTCCTCTTCAC-3 primers from HCT116-SN6 cDNA. The amplicon was digested with *Hind*III and *Hae*III and then cloned in *Hind*III-*EcoR*V digested and dephosphorylated pcDNA3 plasmid. Ligation was transformed in C-Max5αF' cells that were grown on LB with 100 μg/ml ampicillin for 16 h at 37°C. Transformants were selected by PCR screening; PCR products were then purified and sequenced.

### High Resolution Melting (HRM) analysis

Primers were designed using the LightCycler Probe Design Software 2.0 and were: 5'-GATGAGAACATCCCAGCGA-3' and 5'-GCAAGTTCATCATAGACTTCTCAA-3' for the detection of the p.R621H mutation; 5'-CAGTTGATGAAGCTGGAAGT-3' and 5'-CTGTGATCCTAGGGTCCAGAT-3' for the detection of the p.E710G mutation. HRM analysis of cDNA samples or PCR products (amplification of the *TOP1 *region that contains the p.R621H and p.E710G mutations, as described in the previous paragraph "DNA cloning") were carried out in duplicate using the LightCycler 480 System (Roche, Indianapolis, IN, USA). The reaction mixture in a 10 μl final volume consisted of 0.2 μM of each primer, 5 μL of master mix, which contained the Taq polymerase, nucleotides and the ResoLight dye (Roche, Indianapolis, IN, USA), 3 mM of MgCl2 and 10 ng of cDNA. The PCR program consisted of an initial denaturation-activation step at 95°C for 10 min followed by 40 cycles (denaturation at 95°C for 15 s, annealing at 58°C 15 s and elongation at 72°C for 15 s with reading of the fluorescence; acquisition mode: single). The melting program included three steps: denaturation at 95°C for 1 min, renaturation at 40°C for 1 min to encourage heteroduplex formation and melting over the desired temperature range (76-92°C unless otherwise stated) to obtain the HRM curve data at a rate of 25 acquisitions per 1°C. The melting curve analysis carried out by the Gene-Scanning Software comprises three steps: normalization of the melting curves by equalizing the initial fluorescence and the remaining fluorescence after DNA dissociation to 100% and 0%, respectively; shifting of the temperature axis of the normalized melting curves to the point where the entire double-stranded DNA is completely denatured; and finally, analysis of the differences in the melting-curve shapes by subtracting the curves of wild type and mutated DNAs (difference plot).

### Immunocomplex of enzyme (ICE) bioassay

Covalent TOP1-DNA adducts were isolated using the ICE bioassay according to a previously published procedure [24]. Briefly, 10^6 ^cells were lysed with 1% sarcosyl and layered on step gradients containing CsCl solutions (2 mL each) of the following densities: 1.82, 1.72, 1.50, and 1.45. Tubes were centrifuged at 165,000 × g in a Beckman SW40 rotor for 17 h at 20°C and 0.5 ml fractions were collected from the bottom of the tubes. DNA-containing fractions were pooled, normalized for DNA concentration, diluted with an equal volume of 25 mM NaPO4 buffer (pH 6.5) and applied to Immobilon-P membranes with a slot-blot vacuum manifold. TOP1-DNA adducts were visualized by immunostaining using the polyclonal anti-TOP1 antibody from Abcam (ab-3825, Cambridge, MA, USA).

### **Phosphorylated H2AX quantification by flow cytometry analysis**

Cells were seeded in 25 cm^2 ^flasks (2 × 10^5 ^cells/flask). After a 48-hour rest, cells were incubated with 0.5 μM SN38 for 20 hours. One million cells were pelleted, fixed and permeabilized according to the H2AX Phosphorylation Assay Kit (Upstate Biotechnology, Lake Placid, NY, USA) and incubated with the anti-phospho-Histone H2A.X FITC conjugated antibody to detect Histone H2A.X phosphorylation at Serine 139. Analyses were done using an Epics Coulter flow cytometer (Beckman, Ramsey, MN, USA) and results quantified with the Expo 32 analysis software (Coulter, Ramsey, MN, USA).

### Phosphorylated H2AX detection by fluorescence microscopy

Cells growing on chamber slides were treated with 0.5 μM SN38 for 20 hours. Slides were fixed with 4% paraformaldhehyde/PBS at room temperature for 10 min, permeabilized with 0.1% Triton X100/PBS at room temperature for 10 min and blocked with PBS containing 5% BSA at 37°C for 30 min. Slides were incubated with 1/100 diluted mouse anti-phospho-histone H2AX (ser139) FITC conjugated antibody (Upstate Biotechnology, Lake Placid, NY, USA) and DAPI was used to stain the nucleus. Stained cells were mounted in Moviol, and images were recorded using a 63XNA objective on a Leica inverted microscope.

### DNA combing

DNA combing was performed as previously described [25,26]. DNA fibers from the different HCT116 clones were extracted in agarose plugs immediately after labeling and were stretched on silanized coverslips. In fork recovery experiments, CldU and IdU were detected with the anti-BrdU antibodies BU1/75 (AbCys, Paris, France) and BD44 (Becton Dickinson, Franklin Lakes, NJ, USA), respectively. DNA fibers were analyzed on a Leica DM6000B microscope equipped with a CoolSNAP HQ CCD camera (Roper Scientifics, Sarasota, Florida, USA) and a 40x objective. Image acquisition was performed with MetaMorph (Roper Scientifics, Sarasota, Florida, USA). Representative images of DNA fibers were assembled from different fields of view and processed as described [27] with Adobe Photoshop.

### Cell cycle distribution

To determine the cell cycle distribution, SN38 sensitive and resistant, exponentially growing HCT116 cells were plated (1 × 10^5 ^cells) in 6-well plates and 24 hours later cells were exposed to 20 nM SN38. After 24 hours of treatment, untreated and treated cells were washed in ice cold PBS, fixed in 70% ethanol and labeled with 40 μg/ml propidium iodide (Sigma-Aldrich, Saint-Louis, MO, USA) containing 100 μg/ml RNase A (Sigma-Aldrich, Saint-Louis, MO, USA) at 37°C for 1.5 hours. Cell cycle distribution was then determined with a FACScan fluorescence-activated cell sorter (Becton Dickinson, Franklin Lakes, MD, USA) using the FL-2 channel. Cells were gated on a dot plot that displayed DNA pulse-width versus DNA-pulse area to exclude aggregated cells. Cell cycle distributions were illustrated using the WinMDI2.8 histogram analysis software (Phoenix Flow Systems, San Diego, CA, USA) and quantified using Multicycle (DNA-cell cycle analysis software distributed by Phoenix Flow Systems, San Diego, CA, USA).

### Colorectal cancer patients

Normal and cancer colon samples and hepatic metastases were obtained from 45 advanced colorectal cancer patients with synchronous hepatic metastases who were followed at the CRLC Val d'Aurelle, Montpellier, France. The clinical features of these patients have been described elsewhere [28]. All patients underwent surgery for primary tumor resection during which hepatic biopsies were also taken. Treated metastases samples were obtained from patients who were diagnosed with unresectable hepatic metastases and had received CPT-11-based therapy that ended less than 2 months before metastasis resection.

## Results

### Characterization of the SN38 resistant HCT116 clones

To obtain SN38 resistant clones, we exposed the colon adenocarcinoma cell line HCT116 to increasing concentrations of SN38 (Figure 1A). All five resistant clones grew slower than the sensitive HCT116-s clone and consequently their doubling times were 1.2- to 2.1-fold higher than in HCT116-s cells (Figure 1B). The resistant clones showed different in vitro level of resistance to SN38 as indicated by the 6- to 56- fold increase of their IC_50 _value (determined by SRB assay) in comparison to the sensitive HCT116-s cell line (Figure 1B). We have already shown for HCT116-SN50, that high level of resistance to SN38 is associated with over-expression of the ABCG2 efflux pump [7]. This finding was confirmed in the HCT116-A2 clone which displayed a comparable high level of resistance to SN38 (Figure 1C). Conversely, the three other resistant clones did not express ABCG2, suggesting that these clones have developed different drug resistant mechanism. To exclude other efflux mechanisms, the expression of two other efflux proteins known to transport camptothecin derivatives, Pgp and MRP1, was assessed by western blotting. Compared to the MCF7-R positive control, none of the clones displayed any detectable amount of PgP or MRP1 (Additional file 1, Figure S1). We also evaluated by western blotting the expression of Topoisomerase I (TOP1), which is often involved in resistance to camptothecin derivatives. However, its expression (Figure 1C) did not vary significantly in SN38 sensitive and resistant HCT116 cells.

### Resistant HCT116 cell lines contain novel *TOP1 *mutations

**Figure 2:**
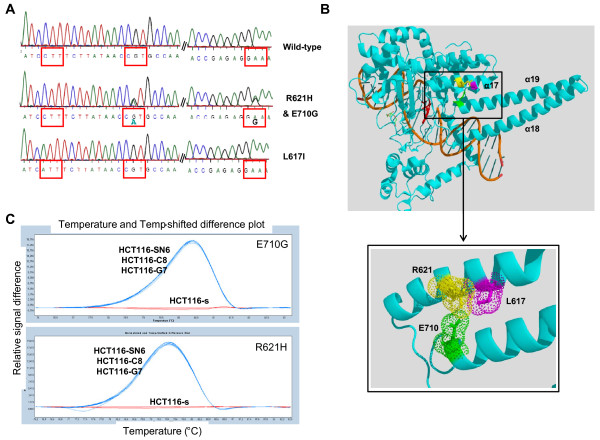
**New *TOP1 *mutations**: A) The heterozygous mutations p.R621H and p.E710G in HCT116-SN6, HCT116-C8, HCT116-G7 cells and the homozygous mutation p.L617I in HCT116-SN50 and HCT116-A2 cells were identified by sequencing of *TOP1*. B) Co-crystal structure of the 70Kd C-terminal portion of human TOP1 covalently linked to DNA and Topotecan (Protein Data Bank entry 1K4T). The three new *TOP1 *mutations are located in the core subdomain III (p.R621H and p.L617I) and in the linker domain (p.E710G). The figure was generated using PyMol [38] and PovRay software [39] C) p.R621H and p.E710G *TOP1 *mutations were detected by HRM analysis in the SN38 resistant cell lines (HCT116-SN6, -C8, -G7), but not in the sensitive HCT116-s clone. The melting profile of HCT116-s was chosen as baseline and the relative differences in the melting of the other samples were plotted relative to this baseline.

We previously partially sequenced the TOP1 gene of the HCT116-s and the HCT116-SN6 cells, and did not find any of the frequently reported mutations leading to camptothecin resistance (p.F361, p.G363, p.R364, p.G503, p.D533, p.G717, p.N722, p.Y723 and p.T729) [22] in both cell lines. However, since new mutations have been recently identified [14], we sequenced the entire TOP1 cDNA of the sensitive and the resistant HCT116 clones. In HCT116-SN6, HCT116-C8, HCT116-G7 cells (moderately resistant clones) we found two heterozygous *TOP1 *point mutations that resulted in missense mutations at position 621 and 710 of the protein. Both mutations were transitions (G→A and A→G) that caused Arginine to Histidine (p.R621H) and Glutamate to Glycine (p.E710G) substitutions (Figure 2A). These two residues interact together via a salt bridge and form part of the interface between helices 17 and 19 (Figure 2B). Both mutations may affect this interaction by two mechanisms. First, the salt bridge is abolished by p.E710G mutation and, since Histidine is only slightly protonated at neutral pH, destabilized by p.R621H substitution. Second, both mutations may also affect the packing of the two helices since they introduce a residue with a shorter side chain. In conclusion, both mutations either present together or alone, may destabilize the interaction between helices 17 and 19 of the enzyme. However, if the two mutations are located on the same allele, the cell will express both a wild type and a mutated form of the TOP1 protein. We thus cloned the *TOP1 *region, which contains the p.R621H and p.E710G mutations, from HCT116-SN6 cDNA and eight individual clones were sequenced. The p.E710G mutation was present in seven clones and the p.R621H only in one. In conclusion, HCT116-SN6, HCT116-C8, HCT116-G7 cells contain two new *TOP1 *mutations which are heterozygous and located on different alleles, indicating that these three cell lines only express mutated forms of TOP1. The presence of both mutations in these cell lines was confirmed by HRM curve analysis, a powerful method for genotyping single nucleotide mutations that is based on the detection of small differences in the PCR dissociation curves. The subtractive fluorescent difference plots of wild type and mutated *TOP1 *DNA allowed a clear discrimination between the homozygous (HCT116-s) and heterozygous (HCT116-SN6, HCT116-C8, HCT116-G7) cDNA samples (Figure 2C). These results were obtained using *TOP1 *primers that surround the p.R621H and p.E710G mutations.

On the other hand, the highly resistant clones HCT116-SN50 and HCT116-A2, which over-express ABCG2, both contained a homozygous mutation (C→A transversion), which resulted in a Leucine to Isoleucine substitution at position 617 of the TOP1 protein (Figure 2A). This mutation is located in helix 17 just one turn before the p.R621H mutation found in the moderately resistant clones. Despite its conservative nature, this mutation could also affect the packing of helix 17 and 19 (Figure 2B).

### Trapping of DNA cleavage complexes is reduced in SN38 resistant HCT116 cells

**Figure 3:**
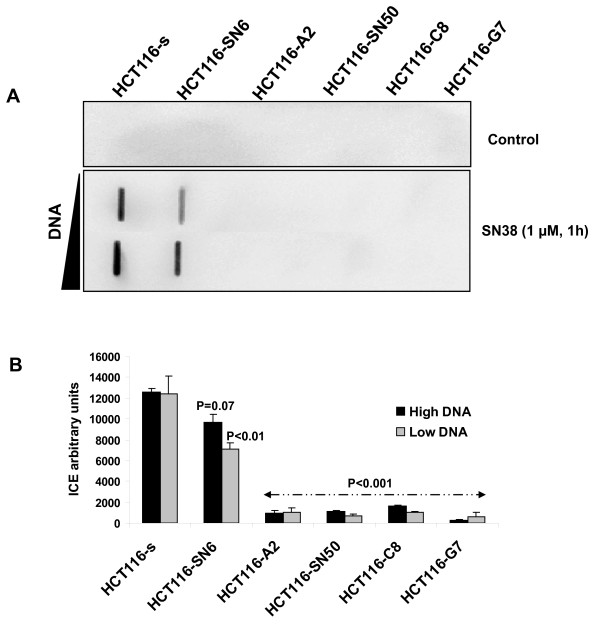
**CPT-11-induced TOP1-DNA complexes**: A) TOP1-DNA cleavage complexes measured using the ImmunoComplex of Enzyme (ICE) assay in nuclear extracts (two concentrations were used) of SN38 sensitive and resistant HCT116 cells following treatment with SN38 (1 μM, 1 h). B) Quantification of TOP1-DNA complexes using Image J Software. The relative intensity of the immune complexes in SN38-treated cells was normalized to that of untreated cells. Vertical bars indicate the SD of three independent experiments. *p*-values less than 0.05 were considered statistically significant.

In a previous study, we found that in HCT116-s and HCT116-SN6 cells, which show similar levels of TOP1 expression, TOP1 had comparable intrinsic catalytic activity by performing a TOP1-mediated DNA relaxation assay using nuclear extracts [22]. Therefore, since SN38 resistance seems not to be associated with changes in TOP1 expression and catalytic activity, we assessed whether the *TOP1 *mutations were functionally relevant by measuring TOP1 cleavage complexes with the ICE bioassay in SN38 resistant and sensitive HCT116 cells treated or not with 1 μM SN38 for 1 hour. Upon SN38 treatment, TOP1 cleavage complexes were strongly induced in HCT116-s cells, whereas all the resistant clones showed much weaker induction (up to 90% lower) whatever the concentration of DNA used (Figure 3A and 3B). This result indicates that trapping of cleavage complexes by CPT-11 is decreased in the resistant cell lines, possibly due to a lower sensitivity of mutant TOP1 to CPT-11 activity.

### DNA double strand break formation is reduced in SN38 resistant HCT116 cells

**Figure 4:**
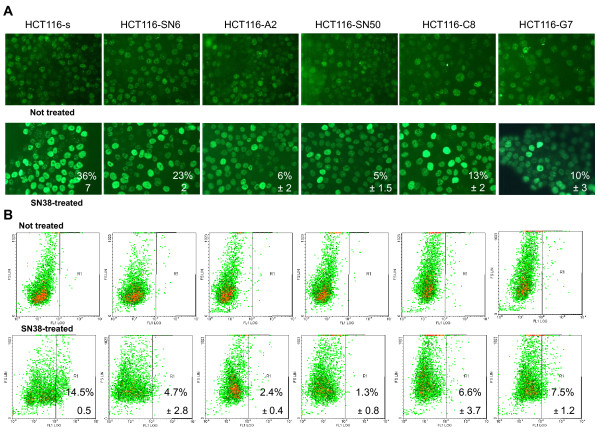
**Assessment of DNA double strand break **formation by measuring H2AX phosphorylation in SN38 sensitive and resistant HCT116 cells treated or not with SN38 (0.5 μM SN38 for 20 hours). A) Immunofluorescence: % indicates the number of cells with entirely stained nucleus. B) Fluorescence-activated cell sorting: % indicates the number of cells with fluorescence intensity >10^2^. Data represent the mean ± SD of at least 3 independent experiments.

The conversion of irreversible TOP1 cleavage complexes leads to DNA cleavage and double-strand break (DSB) formation. Given the strong decrease of CPT-11-DNA-TOP1 cleavage complexes observed in the resistant cell lines, we quantified DSB formation in SN38 sensitive and resistant HCT116 cells, treated or not with 0.5 μM SN38 for 20 hours, by following phosphorylation of H2AX [5] by immunofluorescence (Figure 4A) and fluorescence-activated cell sorting (Figure 4B). In both experiments we observed a decrease in H2AX phosphorylation (gamma-H2AX) in SN38 resistant cells. To compare these results, we quantified the percentage of cells with strong H2AX phosphorylation (i.e., nucleus entirely stained for IF or fluorescence intensity >10^2 ^for FACS experiments). By IF, the percentage of strongly gamma-H2AX-positive cells decreased from 36% in HCT116-s cells to 23% in HCT116-SN6 (moderately resistant clone) and to 6 and 5% in HCT116-A2 and HCT116-SN50 (highly resistant clones) (Figure 4A). FACS quantification confirmed these results (Figure 4B). The percentage of strongly gamma-H2AX-s positive cells decreased from 14.5% in HCT116-s to 4.7%, 6.6% and 7.5% in HCT116-SN6, HCT116-C8 and HCT116-G7 cells, respectively, and to 2.4% and 1.3% in the highly resistant HCT116-A2 and HCT116-SN50 clones. To avoid the impact of repair processes on DSB quantification, the same FACS experiments were performed after a short exposure to SN38 (5 μM, 1 h). We showed that the phosphorylation of H2AX is also reduced in the resistant clones in the similar ratio (50 to 70% decreases) than after SN38 long exposure (Additional file 2, Figure S1). These results demonstrate that DSB formation is reduced in SN38 resistant cells following incubation with SN38, presumably due to the strong decrease in TOP1 cleavage complex formation in these cells (Figure 3).

### *TOP1 *mutations decrease SN38-induced replication fork asymmetry

**Figure 5:**
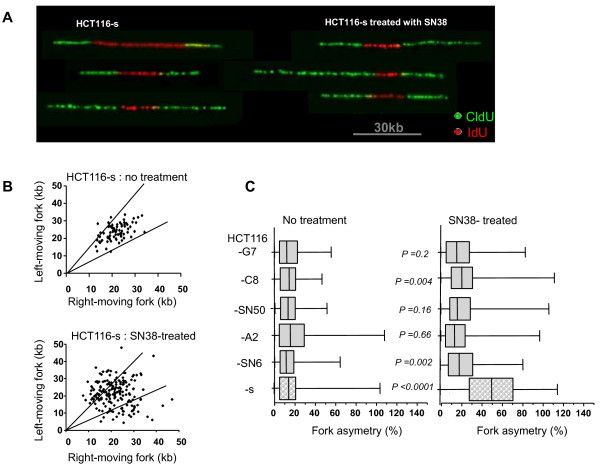
**Analysis of sister replication-fork progression in SN38 sensitive and resistant HCT116 cells **: A) HCT116-s (SN38 sensitive) and HCT116-SN6, -A2, -SN50, -C8 and -G7 (SN38 resistant) cells were pulse-labeled with IdU and CldU and processed for DNA combing. Replication forks progress bidirectionally from the origin and incorporate the analogues. A replication fork arrest is detected as an asymmetrical replication signal. Representative pairs of sister replication forks were assembled from different fields and were centered. Red: IdU and Green: CldU. B) Scatter plot of the distances covered by right-moving and left-moving sister forks during CldU pulse-labeling in HCT116-s treated or not with SN38 (each point represents one measurement). The central areas delimited with lines contain sister forks with less than 25% length difference. The difference of asymmetry is significant p < 0.0001 (Mann Whitney test). C) Box plot of fork asymmetry. Fork asymmetry is expressed as the ratio between the longest and the shortest distance covered by each pair of sister replication forks during CldU pulse-labeling.

Since the primary cytotoxic mechanism of CPT-11 or SN38 in dividing cells is due to collision between trapped TOP1 cleavage complexes and moving DNA replication forks, and TOP1 depleted cells display an increase in forks stalling without any treatment [29,30], we asked whether *TOP1 *mutations in HCT116 resistant cells could influence DNA fork progression (in particular stalling or pausing). We used DNA combing to analyze the progression of sister replication forks in individual DNA fibers from the five SN38 resistant clones and the sensitive HCT116-s cells before and after SN38 treatment. Cells were successively pulse-labeled with IdU (5-iododeoxyuridine) and CldU (5-chlorodeoxyuridine) and the distance covered by right-moving and left-moving sister replication forks during the CldU pulse was determined. Two types of replication signal could be observed: symmetric (equal length of CldU green tracks) and asymmetric labeling (Figure 5A), indicating that replication forks paused or stalled more frequently. In untreated HCT116-s cells, sister forks progressed at a similar rate from a given origin and generated symmetrical patterns with all the values included in less than 25% length differences (Figure 5B). Following treatment with SN38, 50% of the patterns detected in HCT116-s cells were asymmetrical, indicating that the forks stalled more often than in untreated cells probably because of DNA double strand break formation. Analysis of the ratio between the longest and the shortest CldU signals for each pair of sister replication forks revealed a 4-fold increase in fork asymmetry in HCT116-s cells following treatment with SN38 in comparison to untreated cells (% of asymmetry median = 48%, p < 0.0001) (Figure 5C). Conversely, no increase in forks asymmetry was observed after SN38 treatment in the SN38 resistant clones HCT116-SN50, -A2 and -G7, and only a weak increase in HCT116-SN6 and -C8 cells. In the absence of drug, the five resistant clones showed percentage of fork asymmetry comparable to those of untreated HCT116-s cells (percentages of asymmetry median from 11% for HCT116-s to 15% for HCT116-A2) (Figure 5C). This result indicates that, differently from TOP1 depletion, the *TOP1 *mutations identified in the five resistant HCT116 clones have no impact on replication fork progression when cells are untreated [30]. Moreover, the weaker effect of SN38 on replication fork asymmetry in SN38 resistant clones could be linked to the reduced formation of cleavage complexes and DNA DSB in these cells.

### *TOP1 *mutations decrease SN38-induced cell cycle perturbations

**Figure 6:**
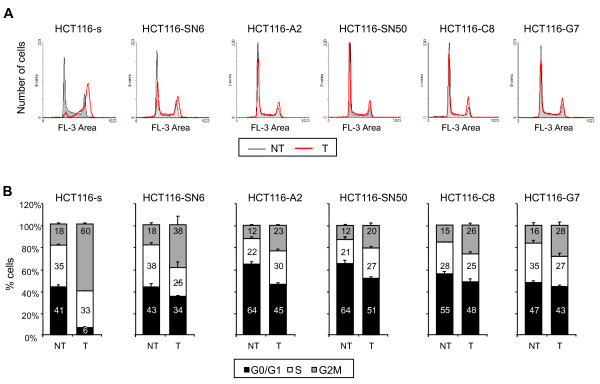
**Effects of SN38 on cell cycle distribution**. A) Cell cycle profiles of SN38 sensitive and resistant HCT116 cell lines treated (T, red line) or not (NT, grey line) with 20 nM SN38 for 24 hours. B) Cell cycle distribution in SN38 sensitive HCT116-s cells and SN38 resistant HCT116-SN6, -S2, -SN50, -C8, -G7 cells with or without treatment with 20nM SN38. The percentages for each cell cycle phase are presented as the mean ± SD of at least 3 independent experiments.

Since G_2 _phase cell cycle arrest is known to be a cellular response to DNA damage, we examined the cell cycle distribution of SN38 sensitive and resistant HCT116 cells following incubation with 20 nM of SN38 for 24 hours. As previously reported, SN38 treatment of HCT116-s cells resulted in strong accumulation of cells in G2/M phase (from 18% to 60%) and in a decrease in the proportion of cells in G0/G1 phase (from 41% to 6%) (Figure 6A-6B). These SN38-induced cell cycle perturbations were less pronounced in the five SN38 resistant cell lines as their cell cycle profiles were similar before and after SN38 treatment (Figure 6A-6B). In the least resistant clone, HCT116-SN6, accumulation in G2/M phase did not go beyond 38% (from 18% in untreated cells) and was only 20% and 23% (from 12% in untreated cells) in the case of the highly resistant clones HCT116-SN50 and HCT116-A2 (Figure 6B). Together these results show that the three identified *TOP1 *mutations decrease the formation of irreversible SN38-TOP1-DNA cleavage complexes, double-strand breaks and replication fork collisions. This leads to a reduction of the drug (CPT-11 or SN38) cytotoxicity, as shown by the higher IC_50 _and the lower effect on cell cycle distribution in all the resistant cell lines.

### **The p.R621H, p.L617I and p.E710G *TOP1 *mutations are not detectable in tissue samples from colorectal cancer patients**

**Figure 7:**
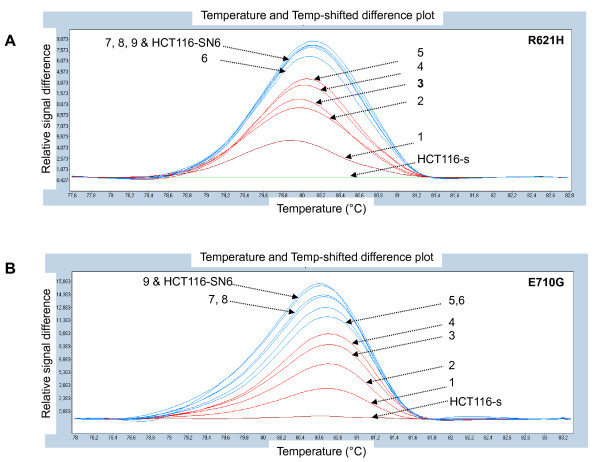
**Assessment of the sensitivity of the HRM analysis**. A) p.R621H and B) p.E710G *TOP1 *mutations detected by HRM in HCT116-s (wild type) and HCT116-SN6 (mutant) cells and in mixtures of wild type and mutant cells: 1 = 10% mutant, 2 = 20% mutant, 3 = 30% mutant, 4 = 40% mutant, 5 = 50% mutant, 6 = 60% mutant, 7 = 70% mutant, 8 = 80% mutant, 9 = 90% mutant.

Finally we investigated whether these three new *TOP1 *mutations were present also in biopsies from patients with colorectal cancer and whether they could be associated with their response to CPT-11. We tested 10 primary tumors (3 responders (R) and 7 non-responders (NR)) and 43 hepatic metastases of which 25 were biopsies before treatment (12 R and 13 NR) and 18 after treatment (12 R and 6 NR) with FOLFIRI (a combination of leucovorin, 5-FU, and CPT-11) We first performed sequencing by the dideoxynucleotide method and we did not find the p.R621H, p.L617I or p.E710G *TOP1 *mutations. However, since the mutation could be present in only a small fraction of cancer cells, we then performed HRM analysis which is known to have a greater sensitivity than the other currently available methods [31]. First, to assess the sensitivity of HRM analysis, serial dilutions of DNA from HCT116-SN6 cells were tested. Each mutation was successfully detected in up to 10% of the cell population (Figure 7). However, in spite of this increase in sensitivity, the p.R621H and p.E710G *TOP1 *mutations were again not detected in any of the samples, either before or after therapy. This indicates that, if these *TOP1 *mutations are present, their frequency is below 10% of the entire cell population and thus impossible to detect with the current methods. As in previous studies [19,20], *TOP1 *mutations seems to be a rare or non-existent event in CPT-11-treated patients.

## Discussion

In this study, we have characterized five SN38 resistant clones derived from the HCT116 adenocarcinoma cell line. They all have a reduced growth rate in comparison to the sensitive HCT116-s cell line in agreement with our previous data on one of the SN38 resistant clones (HCT116-SN6) [22], thus suggesting that their slower growth rate may account as one of the SN38 resistance mechanisms. Moreover, we identified three new mutations in the *TOP1 *gene that could be involved in the cytotoxicity mechanisms of SN38 and CPT-11. Specifically, the point mutations p.R621H and p.E710G are present in moderately resistant clones, whereas the p.L617I mutation is found in the highly resistant clones that also over-express ABCG2.

We then investigated the effects of these mutations on TOP1 and found that, in presence of SN38, both TOP1 cleavage complexes and DSB formation were reduced. Moreover, DNA combing revealed that SN38-induced asymmetry in replication forks was decreased. The asymmetry of sister forks due to the SN38-induced DNA DSB reported here confirms the CPT-11 (or SN38) inhibition of origin firing and fork progression [29]. Indeed, discontinuous fork progression is the consequence of a collision between the replication fork and SN38-stabilized TOP1-cleavable complexes resulting in forks arrest and breakage. Moreover, TOP1 has been recently shown to be involved in the progression of replication forks since TOP1 deficient cells display slower replication fork progression and more pausing or stalling [30]. All these molecular results confirm that in resistant cells treated with camptothecin derivatives there is a decrease of the main mechanism that generates DNA damage that is TOP1 replication-mediated DNA DSB.

The decrease of cleavage complexes could be explained by 1) modified intrinsic catalytic activity of TOP1, inducing decreased rates of DNA cleavage or increased rates of re-ligation and leading to diminution of covalent TOP1-DNA intermediates or 3) reduced affinity of the TOP1-DNA covalent complex for the drug. The first possibility is not likely as we previously showed that TOP1 intrinsic catalytic activity is comparable in the resistant HCT116-SN6 and sensitive HCT116-s cells [22]. Moreover, we show here that the basal level of TOP1 cleavage complexes is similar in all the resistant clones, and they contain an equivalent number of DNA DSB and display similar fork asymmetry than the sensitive cells, showing that TOP1 activity is not affected in the untreated resistant clones. Therefore the most probable mechanism is a decrease in TOP1 affinity for CPT-11 and SN38 due to the mutations. The fact that the three mutations have apparently the same effect can be easily explained by their close proximity in the protein. Indeed, the three residues are packed together, forming part of the interface between helix 17 of the core subdomain III (p.R621H and p.L617I) and helix 19 of the linker domain (p.E710G). Because of this close proximity, we can speculate that whatever the mutation, the effect on the interaction between the core subdomain III and the linker region will be comparable.

Human TOP1 is arranged in four domains: the NH2-terminal domain (residues 1-214), the core domain (residues 215-635), that can be divided in subdomains I, II and III, the linker domain (residues 636-712) and the COOH-terminal domain (residues 713-765). The poorly conserved linker domain, which connects the core and COOH-terminal domains, is highly flexible [32] and is dispensable for the catalytic activity [17]. It has been proposed that the lack of a functional linker accounts for the reduced sensitivity to CPT-11 [33,34]. Among the *TOP1 *mutations known to be involved in CPT-11 resistance only one mutation (p.A653P) was found in the linker domain [18]. The authors suggested that, by increasing the linker domain flexibility, this mutation may alter the conformational changes imposed by drug binding, giving rise to a CPT-11 resistant enzyme. The three mutations described here could confer a resistance phenotype by a comparable mechanism. Indeed, the largest conformational flexibility of TOP1 is between the core subdomain III and the linker domain, which can rotate and shift up to 2.5 Å and 4.6 Å from one to the other. The three mutations are at positions that are involved in the interaction between these two domains but are not disordered in the structure - except slightly in the case of glutamate 710 [32] and could thus be stabilizing residues. As a consequence their mutation could modulate the structural flexibility of the linker and the linker fluctuations could reduce the enzyme affinity for the drug [18,35].

Our knowledge on CPT-11 resistance mechanisms is largely based on in vitro studies. Indeed, several cell lines selected for resistance to CPT-11 have been described and many *TOP1 *mutations that confer resistance to CPT-11 have been identified in these cells (for instance p.F361S, p. R364H, p.E418L, p.G503S, p.D533G, p.A653P, and p.N722S) [17,36,37]. However, such *TOP1 *mutations have never been confirmed in clinical samples. This is also the case of the three new mutations described here. Since we have shown that all the resistant cells with *TOP1 *mutations display reduced growth rate, we could imagine that in a heterogenic tumoral population, cells that carry a *TOP1 *mutation could be overtaken by the sensitive cells as soon as irinotecan selection pressure is lifted. This would explain why it is so easy to identify *TOP1 *mutations in cellular models, but so difficult to confirm them in clinical samples.

## Conclusions

In this report, we describe three new *TOP1 *mutations that are involved *in vitro *in the development of resistance to camptothecin derivatives. These mutations seem to affect TOP1/drug interaction by reducing the affinity or the binding of the drug.

## Competing interests

The authors declare that they have no competing interests.

## Authors' contributions

CG assisted with the design of experiments and drafted the manuscript. NVV performed the HRM experiments and phosphorylated γH2AX quantification by flow cytometry and cell cycle distribution. ST: performed the DNA combing experiments. VD performed Western blots and contributed to cell proliferation analysis. ACa: performed the immunofluorescence experiments and contributed to cell proliferation analysis. CA: performed the ICE experiments. GCB: assisted with the analysis of TopI mutations and with critical examination of the manuscript. ACo: assisted with design of DNA combing assay and with critical examination of the manuscript. PPa: assisted with design of DNA combing assay and with critical examination of the manuscript. PPo: assisted with design of ICE experiments and with critical examination of the manuscript. PM: performed 3D anaysis of TopI and assisted with critical examination of the manuscript. MDR: design the Top1 sequencing, cloning and HRM assay, coordinated the study and drafted the manuscript. All authors read and approved the final manuscript

## Supplementary Material

Additional file 1 **Figure S1: Immunodetection of efflux pumps Pgp, MRP1 **The multidrug-resistant doxorubicin-selected MCF7-R breast cancer cell line was used as a positive control for Pgp and MRP1 [7]. Proteins from the extracts (10^5 ^cells per lane) were electrophoretically separated on 7.5% SDS-PAGE. Primary antibody used were anti-p-Glycoprotein clone F4 (Neomarkers, Fremont, CA) and anti-MRP1 (Alexis Corp, San Diego, CA). Equal loading is shown by β-tubulin (clone tub2.1, Sigma). Click here for file

Additional file 2 **Figure S1: Assessment of DNA double strand break formation **Assessment of DNA double strand break formation by measuring H2AX phosphorylation in SN38 sensitive and resistant HCT116 cells treated or not with SN38. Phosphorylated H2AX quantification was performed by flow cytometry analysis. Cells were plated (1 × 10^5 ^cells) in 6-well plates and 48 hours later, cells were incubated with 5 μM SN38 for 1 hour. Further FACS experiments were performed as described in Methods. % indicates the number of cells with fluorescence intensity >2 × 10^1^. Data represent the mean ± SD of at least 3 independent experiments. Click here for file
