# Supplementary material for: New Topoisomerase I mutations are associated with resistance to camptothecin
Source: Mol Cancer. 2011 May 27;10:64. doi: 10.1186/1476-4598-10-64 (PMC3120799; doi:10.1186/1476-4598-10-64)
Supplement: Additional file 1 — Figure S1: Immunodetection of efflux pumps Pgp, MRP1 The multidrug-resistant doxorubicin-selected MCF7-R breast cancer cell line was used as a positive control for Pgp and MRP1 [7]. Proteins from the extracts (105 cells per lane) were electrophoretically separated on 7.5% SDS-PAGE. Primary antibody used were anti-p-Glycoprotein clone F4 (Neomarkers, Fremont, CA) and anti-MRP1 (Alexis Corp, San Diego, CA). Equal loading is shown by β-tubulin (clone tub2.1, Sigma). [file 1476-4598-10-64-S1.PPT]

## Slide 1
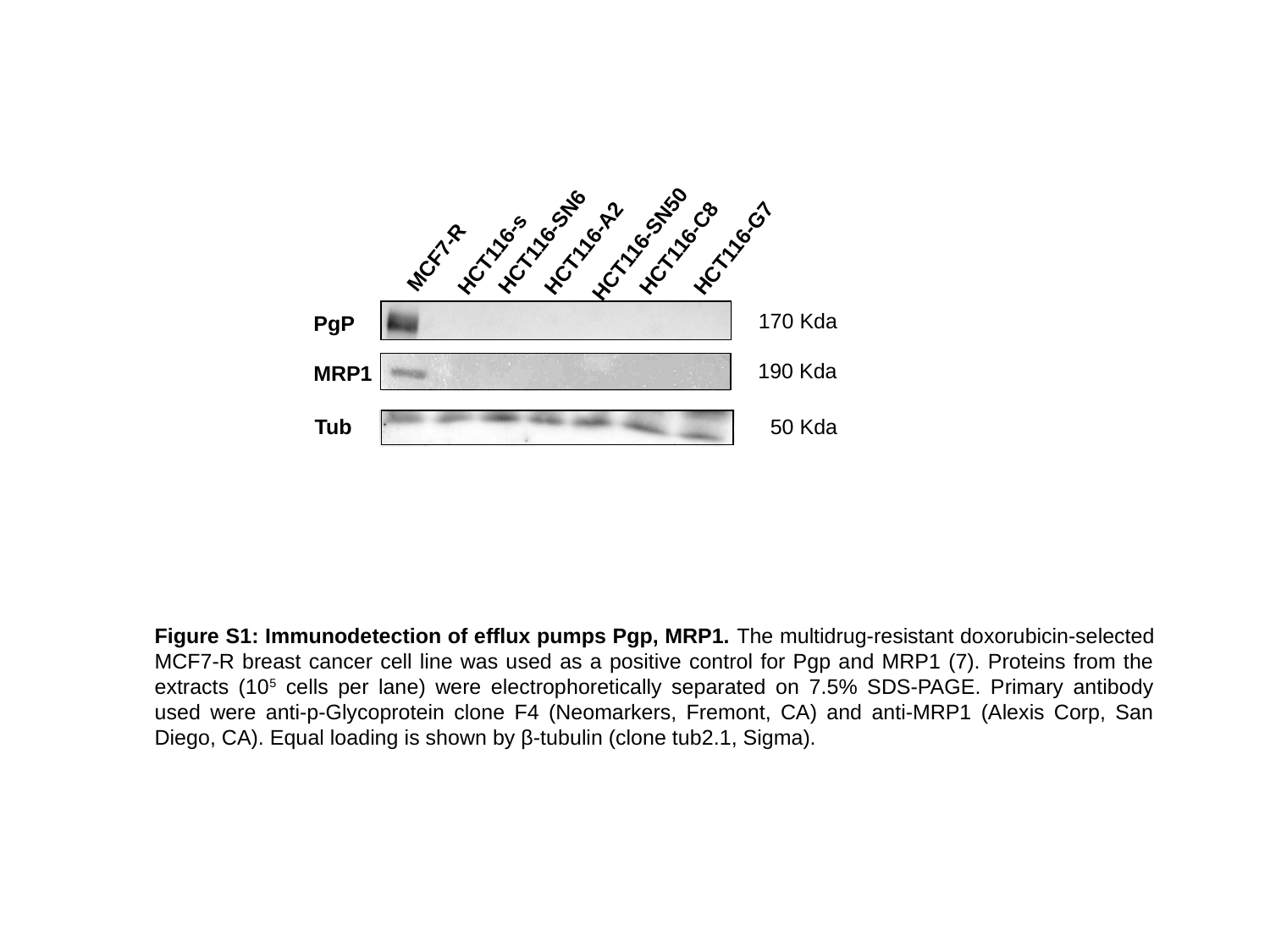

HCT116-SN6
HCT116-SN50
HCT116-A2
HCT116-G7
HCT116-C8
HCT116-s
MCF7-R
170 Kda
PgP
190 Kda
MRP1
Tub
50 Kda
Figure S1: Immunodetection of efflux pumps Pgp, MRP1. The multidrug-resistant doxorubicin-selected MCF7-R breast cancer cell line was used as a positive control for Pgp and MRP1 (7). Proteins from the extracts (105 cells per lane) were electrophoretically separated on 7.5% SDS-PAGE. Primary antibody used were anti-p-Glycoprotein clone F4 (Neomarkers, Fremont, CA) and anti-MRP1 (Alexis Corp, San Diego, CA). Equal loading is shown by β-tubulin (clone tub2.1, Sigma).
