# Supplementary material for: New Topoisomerase I mutations are associated with resistance to camptothecin
Source: Mol Cancer. 2011 May 27;10:64. doi: 10.1186/1476-4598-10-64 (PMC3120799; doi:10.1186/1476-4598-10-64)
Supplement: Additional file 2 — Figure S1: Assessment of DNA double strand break formation Assessment of DNA double strand break formation by measuring H2AX phosphorylation in SN38 sensitive and resistant HCT116 cells treated or not with SN38. Phosphorylated H2AX quantification was performed by flow cytometry analysis. Cells were plated (1 × 10^5^ cells) in 6-well plates and 48 hours later, cells were incubated with 5 μM SN38 for 1 hour. Further FACS experiments were performed as described in Methods. % indicates the number of cells with fluorescence intensity >2 × 101. Data represent the mean ± SD of at least 3 independent experiments. [file 1476-4598-10-64-S2.PPT]

## Slide 1
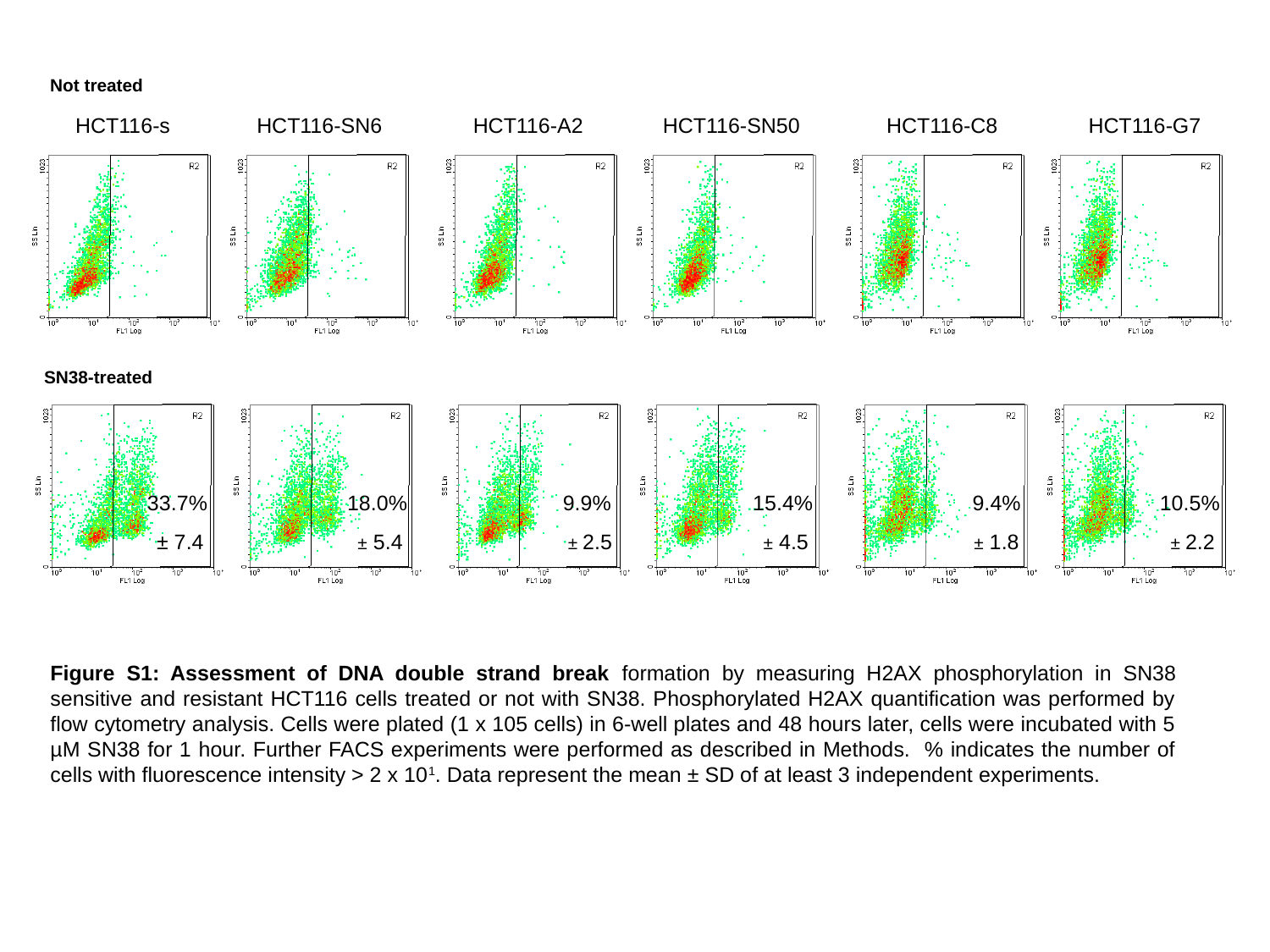

Not treated
HCT116-s
HCT116-SN6
HCT116-A2
HCT116-SN50
HCT116-C8
HCT116-G7
SN38-treated
23%
± 2
13%
± 2
10%
± 3
6%
± 2
5%
± 1.5
36%
± 7
33.7%
± 7.4
18.0%
 ± 5.4
9.9%
± 2.5
15.4%
 ± 4.5
9.4%
± 1.8
10.5%
± 2.2
Figure S1: Assessment of DNA double strand break formation by measuring H2AX phosphorylation in SN38 sensitive and resistant HCT116 cells treated or not with SN38. Phosphorylated H2AX quantification was performed by flow cytometry analysis. Cells were plated (1 x 10^5^ cells) in 6-well plates and 48 hours later, cells were incubated with 5 µM SN38 for 1 hour. Further FACS experiments were performed as described in Methods. % indicates the number of cells with fluorescence intensity > 2 x 101. Data represent the mean ± SD of at least 3 independent experiments.
